# Polymeric Hydrogels as Mesenchymal Stem Cell Secretome Delivery System in Biomedical Applications

**DOI:** 10.3390/polym14061218

**Published:** 2022-03-17

**Authors:** Mia Arifka, Gofarana Wilar, Khaled M. Elamin, Nasrul Wathoni

**Affiliations:** 1 Department of Pharmaceutics and Pharmaceutical Technology, Faculty of Pharmacy, Universitas Padjadjaran, Jatinangor 45363, Indonesia; mia19005@mail.unpad.ac.id; 2 Department of Pharmacology and Clinical Pharmacy, Faculty of Pharmacy, Universitas Padjadjaran, Jatinangor 45363, Indonesia; g.wilar@unpad.ac.id; 3 Global Center for Natural Resources Sciences, Faculty of Life Sciences, Kumamoto University, Kumamoto 862-0973, Japan; khaled@kumamoto-u.ac.jp

**Keywords:** mesenchymal stem cell secretome, polymeric hydrogel, secretome delivery systems, biomedical application

## Abstract

Secretomes of mesenchymal stem cells (MSCs) have been successfully studied in preclinical models for several biomedical applications, including tissue engineering, drug delivery, and cancer therapy. Hydrogels are known to imitate a three-dimensional extracellular matrix to offer a friendly environment for stem cells; therefore, hydrogels can be used as scaffolds for tissue construction, to control the distribution of bioactive compounds in tissues, and as a secretome-producing MSC culture media. The administration of a polymeric hydrogel-based MSC secretome has been shown to overcome the fast clearance of the target tissue. In vitro studies confirm the bioactivity of the secretome encapsulated in the gel, allowing for a controlled and sustained release process. The findings reveal that the feasibility of polymeric hydrogels as MSC -secretome delivery systems had a positive influence on the pace of tissue and organ regeneration, as well as an enhanced secretome production. In this review, we discuss the widely used polymeric hydrogels and their advantages as MSC secretome delivery systems in biomedical applications.

## 1. Introduction

**Figure 1:**
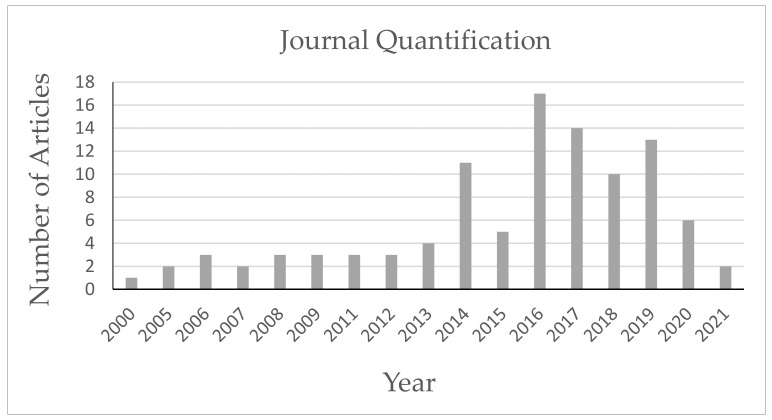
Quantification of journals in this review.

Currently, many chronic diseases of inflammatory and/or degenerative origin associated with several systemic diseases, such as lupus, diabetes, psoriasis, and rheumatoid arthritis, involve the syndrome of aging and debility, which does not receive satisfactory treatment. Cellular therapy with mesenchymal stem cells (MSCs) offers a novel therapeutic approach based on several pharmacological effects, including anti-inflammatory, immunomodulatory, regenerative, pro-angiogenic, and anti-fibrotic properties [1]. MSCs can be obtained from a variety of adult and fetal tissues that are normally disposed of as waste, including adipose tissue, placenta, umbilical cord [2], dental tissue, bone marrow, and menstrual fluid [3] and Wharton’s jelly [4]. Of all the sources, stromal tissue, bone marrow, and subcutaneous fat MCSs are preferred for separation and growth [5,6]. However, many stem cell therapy protocols are limited due to the increased number of cells required and poor cell viability after long-term transplantation [7], while other investigations have found that transplanted cells have low viability and survival in host tissues, with less than 1% of the transplanted MSCs remaining in the target tissue for a long period of time [8,9]. It is known that the therapeutic effect of transplanted MSCs is not the result of the cells themselves, but is related to their ability to secrete bioactive molecules [10]. The complex bioactive molecules, including growth factors, cytokines, chemokines, hormones, and extracellular vesicles, referred to as secretomes, provide a favorable microenvironment for injured tissues, helping to limit the area of damage and to promote regenerative responses [11,12]. There is a growing interest in research related to the biomedical applications of the secretome. A Scopus search (www.scopus.com, accessed on 24 April 2021) with the term “Secretome” yielded 369 publications, and most were published after 2011 (Figure 1).

Secretome-based therapies for tissue repair and regeneration are allogeneic and can be pre-prepared as ready-to-use therapies for a variety of conditions, similar to vaccines and monoclonal antibodies. Moreover, when compared to cell-based products, secretomes can be freeze dried or lyophilized for convenient storage and delivery [13,14]. Secretomes have shown therapeutic effects in animal models of various pathological conditions, such as myocardial infarction [15], tissue ischemia [16], corneal wound healing [17], osteoarthritis [18], Parkinson’s disease [19], acute lung injury [20], liver disease [21], and kidney disease [22]. Secretomes can be considered as a protein-based biotechnology product, which may be safer than products containing live cells [23]. In addition, the secretome can be stored safely for a long period of time, without the risk of losing its biological function. Furthermore, secretomes have a lower risk of tumorigenesis, can be sterilized, and can be treated as active pharmaceutical ingredients (APIs). In addition, the ability to combine them with biomaterials makes secretome therapy a more clinical therapeutic strategy [24,25].

The direct injection of secretomes has been shown to create secretome instability in vivo, as the secretome might be destroyed by enzymes or seep into adjacent tissues [9]. A study proved that the effect of the secretome decreased over time when it was administered in a mouse model for the treatment of Parkinson’s disease, possibly due to its in situ consumption. In a tissue engineering-based approach, one possible strategy to overcome this limitation is the local injection of MSCs and/or MSC secretomes embedded with hydrogels, allowing the formation of long-lasting secretomes with improved cell fate control [19]. Polymer hydrogel-based delivery systems in 3D cultured MSCs, such as scaffolds, could be an important strategy, as they impact cell physiology, increasing the extracellular matrix (ECM) and integrin expression, while promoting secretome secretion [26]. However, the effect of mechanical properties and surface properties of polymeric hydrogels on cell organization and functionality in the distribution of the ECM and secretome molecules, both in culture and direct injection, is still not known, in terms of its effectiveness in biomedical applications [27]. Simultaneously, a large number of studies have focused on secretome delivery systems to overcome the limitations of secretome application through the application of secretome dosage forms contained in polymeric hydrogels. Finally, a perspective view is given regarding the delivery strategy of a polymer hydrogel required for the controlled release of MSC secretomes as a pharmaceutical active ingredient. Based on the above considerations, we review in this paper the use of polymeric hydrogels as systems that facilitate secretome delivery in various biomedical applications.

## 2. Overview of Mesenchymal Stem Cells (MSCs)

MSCs are multipotent cells that are excellent candidates for cellular therapy because of their ability to influence the tissue microenvironment via extracellular vesicles [28]. MSCs, as multipotent adult stromal cells, have self-renewal abilities (that is, they can produce more MSCs on their own) and differentiation potential (into other cell types) [29]. MSCs can differentiate into adipocytes, chondrocytes, osteoblasts, and myocytes, depending on their origin [30,31,32].

MSCs have immunomodulatory abilities that regulate dendritic cells, lymphocytes, macrophages, mast cells, neutrophils, and natural killer cells, which are all involved in the immune response used to treat various inflammatory diseases [6]. MSCs are considered as “ideal materials” for regenerative medicine, because their therapeutic potential was once thought to depend on their differentiation ability [33]. The therapeutic potential of MSCs is thought to be based in part on the release of molecules with unique bioactive soluble factors with paracrine action, which are partly created by the vesicular system, namely the secretome. Secretomes (cell-secreted factors) can pass through the endothelial barrier, enter the bloodstream, and eventually reach injured cells [29]. Furthermore, the production of paracrine growth factors and cytokines helps in the repair of damaged tissues [34,35]. On this foundation, MSCs have been highlighted as effective cell treatments for various diseases in recent decades.

### 2.1. MSCs as a Secretome Source

The bioactive factors from MSCs were harvested and cultured in vitro and called secretome or conditioned medium (CM). The MSC-derived secretome contains a wide variety of pro-regenerative factors (a mixture of secreted factors, including soluble proteins, free nucleic acids, lipids, and extracellular vesicles), MSCs as a new type of regenerative medicine through non-invasive therapeutic cellular approaches so that it can overcome the poor decomposition of transplanted cells [5]. MSCs can self-renew and differentiate into various tissue-specific cell types, which can promote angiogenesis, suppress the immune system, by secreting and remodeling the ECM. MSCs exhibit reparative, regenerative and immunomodulatory effects through paracrine signaling to generate AKT-MSC activity and release various anti-apoptotic, mitogenic, and pro-angiogenic factors as the main mechanism for biomedical applications [36,37]. The role of paracrine is now considered as one of the main features for MSC secretome-mediated repair and regeneration following several in vitro and in vivo studies [38]. Various studies have shown that the MSC secretome has been successfully assessed in various pathological conditions, such as alopecia, acute and chronic wound healing, myocardial infarction, acute liver injury, cerebral and spinal cord injury, lung injury, and bone defects [39]. On the other hand, the standardization of soluble factor production methods from secretome MSCs will allow the development of off-the-shelf treatments that no longer require long preparation processes by isolating and spreading autologous cells in vitro from patients [40].

### 2.2. Secretome

**Figure 2:**
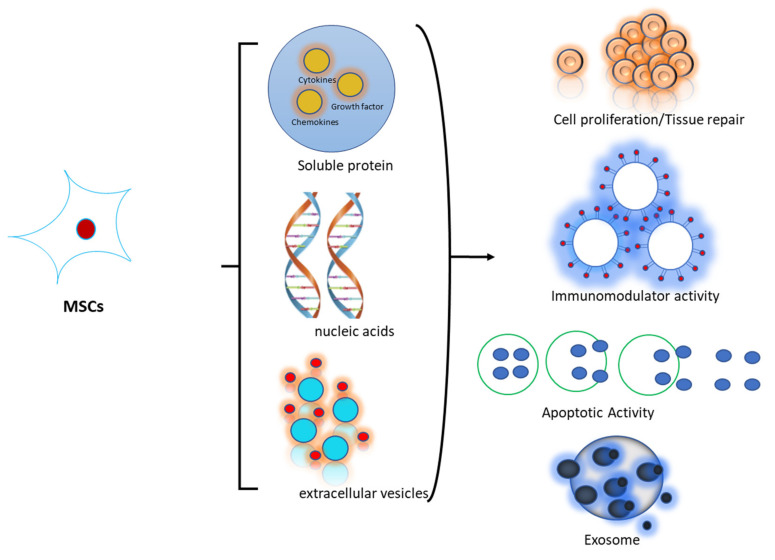
Scheme of the Mechanism of Action of MSCs.

Secretome or CM is defined as a set of bioactive factors secreted by stem cells into the extracellular space, which includes soluble proteins (e.g., cytokines, chemokines, and growth factors), lipids, free nucleic acids, and membrane-bound extracellular vesicles containing biomolecules, such as apoptotic bodies, microparticles, and exosomes, used as a non-cellular therapeutic approach [41,42]. In fact, secretome products isolated from MSCs can effectively mimic the therapeutic effects of MSCs in preclinical models. Stem cell secretomes show promising results in tissue repair (such as heart, nerve, liver, lung, kidney, and skin), including proangiogenic, antiapoptotic, antifibrotic, anti-inflammatory, and immunomodulatory effects [43,44]. Neither the entire CM nor the extracellular vesicles obtained from cultures of MSCs of different human origin confer broad therapeutic benefits (Figure 2). Indeed, all cells release a variety of bioactive molecules that reflect their functional state as well as the effect of their surrounding milieu. The number and variety of released bioactive factors vary as MSCs enter and progress toward an end-stage phenotype, and as MSC progeny enter new lineage stages. The pattern and quantity of such released components are widely understood to feed back on the cell and regulate both its functional status and physiology [10].

Secretomes represent communication pathways between cells and play important roles in several cellular mechanisms, such as the exchange of genetic material, transfer of biologically active molecules, and defense against viral attack in mammalian cells [25,45]. MSC-secreted bioactive compounds can have direct, indirect, or even both effects: direct by generating intracellular signaling, or indirect by influencing another cell nearby to produce the functionally active chemical. This indirect activity is referred to as trophic. Bioactive factor functional secretions (paracrine and autocrine) can have a big impact on local cellular dynamics. The marrow stroma formed from MSCs, for example, not only offers the informational connective tissue floor space for cell anchoring, but it also serves as a massive learning tool for both vascular and hematopoietic cells [10]. The MSC secretome has shown therapeutic effects in animal models of various pathological conditions, such as myocardial infarction, stroke, tissue ischemia, spinal cord injury, wound healing, arthritis, Parkinson’s disease, musculoskeletal disease, acute lung injury, liver disease, and kidney disease [9].

The therapeutic efficacy of secretome is related to three key mechanisms of action: (i) the homing ability, in which systemically administered cells selectively migrate to injured tissue via chemoattraction mediated by cell surface receptors of chemokines, integrins, and selectins; (ii) the ability to differentiate into new, different cell types to replace damaged cells; and (iii) the ability to secrete substances that can modulate resident cell responses via paracrine action [46,47]. Secretomes can establish communication between cells, and their regenerative, anti-inflammatory, and other abilities are critical in restoring physiological balance in injured cells and, by extension, throughout organs. This intercellular communication may be mediated by newly discovered microanatomical fluid-filled gaps within and between tissues [13,48].

The use of cell-free therapies in regenerative medicine, such as MSC-sourced secretions, has several advantages over stem cell-based applications: (a) the use of secretomes eliminates some of the potential safety concerns associated with the transplantation of live cell and population proliferative cells, such as immune compatibility, tumorigenicity, embolism formation, and transmission of infection; (b) secretomes sourced from MSC can be evaluated for safety, dosage, and potency in the same way as conventional pharmaceutical ingredients; (c) storage can be carried out without the use of potentially toxic cryopreservative agents for extended periods, without loss of product potency [17,49,50]; (d) due to the avoidance of invasive cell collection techniques, using MSC-sourced secretomes is more cost effective and practical for clinical applications [51]; (e) mass production is possible under controlled laboratory conditions using tailor-made cell lines, which provide a suitable source of bioactive factors; (f) the time and cost to develop and maintain cultured stem cells can be greatly reduced, and ready-to-use secretome therapies can be made available for the treatment of acute conditions; (g) biological products used for therapeutic purposes can be modified to provide the required cell-specific effects [1,52,53].

### 2.3. Secretome Composition

Studies show that the efficacy of MSCs for biomedical applications is mediated by secreted factors, such as insulin-like growth factor 1 (IGF-1), vascular endothelial growth factor (VEGF), transforming growth factor beta-1 (TGF-β1), basic fibroblast growth factor (bFGF), placental growth factor (PGF), nerve growth factor (NGF), interleukin-1 (IL-1), IL-6, IL-10, and Tumor Necrosis Factor alpha (TNF-α) [36,38,50]. The secretomes of different MSC populations in different tissues appear to be variable, and their composition may change in response to physiological and pathological situations [23]. MSCs generated from adipose tissue were found to have high levels of VEGF-D, IGF-1, and IL-8 mRNA transcripts [51]. MSCs generated from the placenta showed increased levels of expression of hepatocyte growth factor (HGF), bFGF, IL-6, IL-8, IL-1α, and IL-1β, whereas populations of MSCs generated from bone marrow were found to produce VEGF-A, NGF, and large amounts of angiogenin [52].

The secretome released by MSCs can vary depending on a number of parameters, including cell species, tissue source, isolation technique, chemical and physical stimuli, and the cell microenvironment. Different secretome profiles and angiogenic potentials can be found in MSCs derived from different sources. Adipose-derived stem cell (ADSC) MSC secretome has a greater range of angiogenic factors than bone marrow stem cells (BMSCs) for angiogenesis-mediated tissue regeneration condition [51,53]. Because of their greater potential to induce neuronal axon growth, ADSC secretome may be favored for neuroregenerative applications [54]. Compared with BMSCs, the MSC secretome of Wharton’s jelly is more suitable for neurogenesis and angiogenesis [55]. Endothelial progenitor cells, which are one of the most significant cell types in neovascularization, respond differently to MSC secretomes separated from the placenta and bone marrow. While the MSC secretome from the placenta promotes endothelial progenitor cell migration, the BMSC secretome has a greater impact on cell invasion and vascular formation [56]. This distinction emphasizes the importance of considering several factors when selecting a cell source for secretome isolation.

## 3. Biomedical Applications of Secretome

The reported actions of the MCS secretome products following local injection in numerous experimental in vivo models are undoubtedly the most plausible scientific evidence of their biological effects [25]. The entire conditioned media or extracellular vesicles (EVs) derived from various human origin MSC cultures provide significant therapeutic effects that are associated with biomedical applications, such as bone regeneration, and in the treatment of cardiovascular diseases, neurological diseases, and kidney disorders [53,54]. Many studies have shown that secretome-derived products of MSCs, such as exosomes and conditioned media (CM), have therapeutic effects, such as cell differentiation and proliferation, angiogenesis, and vasculogenesis [41]. Most of the soluble bioactive factors produced by MSCs exhibit strong angiogenic effects (VEGF, HGF, FGF, and IL-6), anti-apoptotic effects (VEGF, HGF, FGF, and IL-6), immunomodulatory (HGF, IL-6), or neuroprotective ((brain-derived neurotrophic factor (BDNF), NGF)) [55,56]. Such MSCs are known to represent therapeutic products that are nearly ready for use in the most common situations. In biomedical applications, the use of MSC-secreted secretomes is thought to be associated with a lower risk than the use of therapeutic products containing live cells [39,57]. Some secretome applications in biomedicine can be seen in Table 1.

## 4. Secretome Delivery Systems in Biomedical Applications

In general, secretome administration is considered safer than live cell transplantation of MSCs. In fact, this leads to a lower risk of embolic formation after intravenous infusion and a lower risk of tumorigenic or pathological transformation due to uncontrolled cell differentiation. In addition, because the secretome reflects the composition of the stem cell, it can retain the same distinctive immune properties as the MSCs, allowing the use of allogeneic preparations (even between species) without immune activation. Another important advantage to consider is that treatment with a secretome is not permanent. Regarding safety, although high doses are administered in some cases, no side effects have been reported; thus, if any side effects are present, they can be interrupted more easily than with cell administration. Doses and routes of administration vary substantially in different pre-clinical animal studies; therefore, a safe and effective dose must be determined for use in the treatment of these pathological conditions. In addition to the right dose, the appropriate time and frequency of administration also need to be determined by further research. In fact, secretome may be administered in repeated doses to maintain its therapeutic potential over time, as several authors have reported [20,39,77].

Secretome delivery in biomedical applications is quite difficult, mainly because the damaged tissue is not easily targeted. Various delivery systems and strategies have been developed to exploit the therapeutic potential of the stem cell secretome in organ/tissue repair and regeneration. Depending on the target site, the secretome or CM can be administered by direct injection, systemic injection, or a delivery device, such as a hydrogel [78].

### 4.1. Hydrogels as Secretome Delivery Systems

Hydrogels are water-soluble polymer networks that can absorb more than 20% by weight in water, while maintaining their three-dimensional structure. Hydrogels have been extensively researched in therapeutic applications, such as tissue engineering and drug delivery, because of their easily modifiable chemical and physical properties, as well as their unique feature that they can be made from any hydrophilic polymer [79]. Hydrogels can be developed as a secretome delivery strategy for controlled and sustained release, thereby prolonging the secretome residence time and providing a safer and more effective therapeutic effect, which, more importantly, does not produce systemic or local problems [80]. One of the earliest works of secretome delivery was based on the development of injectable hydrogels, providing a minimally invasive means for local delivery to target organs. The peptide-based hydrogel sponge that can be injected in situ absorbs the secretome and then releases it in a therapeutic environment [12]. Silk-based injectable hydrogel was used to deliver human umbilical cord MSC secretomes into the bone marrow of osteoporotic mice. In vitro, this hydrogel produces a gradual and continuous release for 30 days. Intratibial injection concentrates the secretome at the site of osteoporosis within the bone, allowing it to exert an anti-aging effect and prevent significant bone loss [66]. An injectable hydrogel made of gelatin and phyllosilicate (LAPONITE)^®^ was made to treat myocardial infarction in rats due to its strong protein adsorption capacity and contribution to the shear-thinning characteristics of hydrogels. Secretomes from spheroid cultures of human ADSCs were introduced by intramyocardial injection and administered to the peri-infarction area of the heart. Secretome delivery improved cardiac function and vascularity, while decreasing fibrosis and scar tissue development [15].

Researchers in academia and business have been working on the design and development of “smart polymers” in general, and hydrogels in particular, in response to a wide range of therapeutic issues and improving treatment standards [81]. Hydrogel-based polymers are known to interact with the biological environment in pre-programmed ways, displaying changes in some of their properties (e.g., viscosity) in response to pH, temperature, electric and magnetic fields, and other factors [82,83,84]. The use of natural biodegradable polymers, such as hyaluronic acid (HA), alginate, gelatin, and carrageenan (CG), as well as synthetic polymers, such as polyester and polyacrylamide, can be an important strategy to control the release and delivery of the secretome. The use of biomaterials can be an important strategy, as in the 3D culture of MSCs in scaffolds or hydrogels that impact cell physiology by increasing the endogenous ECM and integrin expression, while promoting secretome secretion [26]. It is versatile enough to be added to a number of delivery systems that improve the control of secretome release in biomedical applications (Table 2).

### 4.2. Hydrogel-Based Polymerics as Secretome Carriers

Recent research has discovered that hydrogel polymerics have chemical, physical, and biological properties that are similar to ECM in natural tissues, bringing cells together and controlling tissue structure, regulating cell function, and supporting cell attachment, proliferation, and differentiation.The outstanding properties of the hydrogel three-dimensional (3D) hierarchical structure, which allows cells to accumulate and grow inside, organizes cells into a 3D functional tissue, and enables the efficient mass transfer of nutrients, oxygen transfer, and waste exchange in cells and tissues, have resulted in their excellent performances in a variety of biomedical applications [86].

Many different material hydrogels have been explored and used as drug delivery in tissue engineering, ranging from natural biomass to synthetic biopolymers. High elasticity and flexibility, as well as exceptional biocompatibility and biodegradability, are all characteristics of these materials. Natural biopolymers are produced by all organisms during their growth cycles and are frequently available in considerable numbers. Before tissue engineering became a research area, most natural biopolymers were well known in biomedical applications. The group includes proteins, such as collagen and fibrin, polysaccharides, such as chitosan (CS) and hyaluronic acid (HA), and some derivatives of the extracellular matrix. The inherent bioactivities of these natural biopolymers can promote cell adhesion, proliferation, and tissue recovery. The hydrogel could also be constructed from synthetic biopolymers. A commonly used synthetic biopolymer is poly(lactic acid) (PLA), an aliphatic polyester, which could degrade within the human body to form lactic acid, a naturally occurring chemical that can be easily removed from the body. Similar materials include polyglycolic acid (PGA), polycaprolactone (PCL), and poly(lactic-co glycolic acid) (PLGA). Synthetic biopolymers have a long history of use in biological applications and are generally regarded safe. However, numerous disadvantages remain, such as low hydrophilicity, bioinert characteristics, and the lack of biological signals in the ECM, which severely limit their effectiveness in tissue engineering applications. Thus, developing highly bioactive hydrogels to replace, modify, or sustain injured tissues is critical, which can be rationally accomplished by including secretomes into hydrogel systems [87].

#### 4.2.1. Hyaluronic Acid (HA)

HA, also known as hyaluron, is a non-immune polysaccharide containing the units a-1,4-Dglucuronic acid (GlcUA) and b-1,3-acetyl-D-glucosamine (GlcNAc) [88]. HA is a non-sulfate, non-immunogenic anionic glycosaminoglycan distributed throughout the body, in connective tissue, synovial fluid, organs, and the ECM of cartilage [89]. More importantly, HA is a valuable component of the ECM, as it regulates wound healing, cell signaling, angiogenesis, matrix organization, and morphogenesis. One study group found that hMSC differentiation in HA-based hydrogels only depended on degradation-mediated cellular traction [90,91]. The implantation of stem cells into the HA hydrogel affects the release of cytokines/chemokines, thereby influencing the modulation of macrophage immune responses, including macrophage polarization and the release of anti and/or pro-inflammatory molecules [63]. Recently, it was shown that culturing human adipose stem/stromal cells (ASCs) with human chondrocytes in a HA hydrogel reduces the expression of type X collagen as an anti-angiogenic and anti-hypertrophic, and COMP, a biomarker of chondrogenesis, is upregulated in spheroid ASCs. The secretome compositions of induced and non-induced ASCs were compared, and 138 proteins directly relevant to chondrogenesis were found, out of a total of 704 proteins. Scaffold and serum-free techniques offer the development of biomarkers and regenerative treatment procedures that are stable in mimicking cartilage [75].

The use of HA gel as a high-quality antiadhesive biomaterial for controlled release via intrauterine administration can restore endometrial morphology and function after electrocoagulation injury in mice. This therapeutic platform uses paracrine signaling with efficacy similar to that of cell therapy. Adhesion protection allows a fast recovery process using MSC secretomes [24]. The controlled secretome release of rat adipose tissue-derived MSCs (RAAMSCs) containing hydrogels is also known to enhance the disease toxicity mechanisms of Parkinson’s in both in vitro and in vivo models. The secretion of RAAMSCs encapsulated in PEG2000/collagen and collagen/HA gel allowed the restoration of metabolic activity and had a complete protective effect on cells, and the quantification of cell proliferation was not significantly different [19]. The MSC secretome contained in a combined HA/chondroitin sulfate gel in topical eye drops can provide the advantage of a highly consistent treatment formulation, and using a thick gel can reduce the number of topical applications required per day, thus making the treatment more efficient in terms of the volumetric losses incurred compared to the typical loss of standard eye drops [62]. In addition, it has also been shown that stem cell sponge-like hydrogels containing HA hold promise for the enhancement of diabetic wound healing, by promoting reepithelialization and regulating the inflammatory response towards resolution, which appears to be a key factor in successful neo-innervation [64].

The physical properties of the HA hydrogel can be changed to regulate the administration of MSC CM as an injection or implant material for the repair and regeneration of soft and hard tissues [85]. In lipopolysaccharide (LPS) injections, the instillation of a HA hydrogel in EPCs and MSC secretions reduced serum creatinine levels more effectively than nonhypoxic or hypoxic MSC administration 24 h later. Embedded HA-hydrogel alters the EPC and MSC secretomes. It appears that the changes induced by embedding a HA hydrogel in EPC and MSC secretions are a result of the activation of cell surface integrins and intracellular cytoskeleton properties due to the enhanced rigidity (elastic modules) offered by embedding HA hydrogels [63].

#### 4.2.2. Alginate

Alginate is a naturally occurring anionic polymer electrolyte made from polysaccharides derived from brown algae. It is a tight linear copolymer consisting of the monosaccharides a-L-guluronic acid (G) and b-D-mannuronic acid (M). It has been discussed in many published studies because of its abundance, low cost, and biocompatibility, and its application has increased in recent years [92,93]. Alginates have the property of forming a solid three-dimensional structure, dubbed the “egg box”, when interacting with divalent ions, such as Ca^2+^, Sr^2+^, and BA^2+^ to improve mechanical characteristics. However, controlling the rate of degradation may be difficult; generally, cationic polymers have significant problems in this regard. Natural materials have a number of disadvantages, including poor mechanical characteristics and cell adhesion [92]. MSCs in hydrogels have previously been shown to undergo chondrogenesis. Culture in alginate not only prevents chondrocytes from de-differentiating, and even restores the cartilaginous phenotype to differentiated chondrocytes, but also facilitates the synthesis of cartilage-specific molecules, such as proteoglycans and col-2. Moreover, the alginate culture system is suitable for the investigation of cell–cell communication and interaction due to the ease of manipulation of cell concentrations in this 3D system. Together, the 3D alginate culture system combined with the culture of two cell types can be an effective method to enhance chondrogenic differentiation [71].

Aggregate hMSCs incorporated into an RGD-alginate hydrogel can maintain its viability and structure, as well as the formation of the ECM. The morphology of the ECM regions embedded in the alginate fragments was observed, with single cell delivery resulting in a more even distribution of pockets across the construct and aggregate delivery resulting in greater fragmentation, whereas single cells remained trapped in the alginate at 14 days after implantation [73]. Substrate stiffness plays a role during folliculogenesis, because the follicles are subjected to compressive forces from the environment as they develop in the alginate. However, in the 3D alginate culture system, the encapsulated follicles were unable to develop new blood vessels and increase nutrient supply, as would occur in vivo in response to all secreted angiogenic factors. New generation culture systems that incorporate a degradable matrix and allow co-encapsulation of endothelial cells and other cells with growing follicles will overcome some of these limitations in the future [22].

#### 4.2.3. Carrageenan (CG)

CG is a sulfated hydrophilic polysaccharide obtained from various species of red algae and is widely used by the food industry and in regenerative medicine as scaffolds and controlled release systems for the delivery of pharmaceutical drugs, growth factors, and cells. Considering its potential, we previously used CG hydrogels in conjunction with skin-derived MSCs (SD MSCs) to treat skin lesions in a murine wound healing model. The combination of CG and SD MSC is able to reduce inflammation, accelerate recovery, and increase ECM deposition in the wound area. However, the results showed that the use of CG hydrogel or polyvinyl alcohol (PVA), as well as delivery of the embedded SD MSC secretome, did not improve wound healing or lead to better outcomes of tissue vascularization in an in vivo model [7].

#### 4.2.4. Gellan Gum (GG)

A linear anionic microbial polysaccharide named gellan gum (GG) consists of repeating units of glucose, glucuronic acid, and rhamnose. The use of GG and its derivatives for hydrogels has been shown to be beneficial in disc repair/cartilage regeneration. In addition, it has previously been shown that the modification of a hydrogel with an extracellular matrix derived peptide GRGDS-fibronectin can be immobilized onto GG hydrogels. This peptide-modified gellan gel can increase the adhesion and proliferation of neural stem progenitor cells (NSPCs) more than the control GG. The results revealed that the presence of the peptide increased the proliferation of spinal cord cells (BM MSCs), and increased metabolic activity and cell morphology. It was also shown that GRGDS-GG positively modulates BM MSC secretion, which enhances the survival and differentiation of primary cultures of hippocampal neurons in vitro [27].

#### 4.2.5. Collagen

Collagen is a key component of ECM proteins found in mammalian tissues. Natural collagen has been widely used as a highly adaptable biocompatible, biodegradable material and as a biomaterial in tissue engineering. Collagen has a unique helical structure in which three left-handed polypeptide helices intertwine to form a right-handed triple helix, and peptide bonds cross-link adjacent helices at the ends of each helix [94]. As a result, physical and chemical approaches can be used to bind collagen. Physical crosslinks can be used to make reversible hydrogels, but they have poor mechanical qualities. Chemically produced hydrogels, on the other hand, have superior physical qualities.

Previous studies have reported the successful retention and controlled release of VEGF by surface adsorption of bioactive phyllosilicate (LAPONITE)^®^ via electrostatic interactions. The VEGF/phyllosilicate (LAPONITE)^®^ complex was then encapsulated in a collagen scaffold, showing increased angiogenesis in vivo. The nanocomposite hydrogel platform can offer sustained release in the synthetic clay, allowing the full therapeutic benefits to be achieved. This method is recognized as an alternative strategy to the stem cell transplantation approach, producing more paracrine factors [67]. In an in vivo study, compressed collagen had sufficient regenerative ability to induce bladder wall tissue regeneration. This study, seeded with a combination of smooth muscle cells (SMC) and SMC-like adipose-derived stem cells (pADSC), could be a first step towards functional bladder improvement, especially in patients with end-stage bladder disorders [76].

#### 4.2.6. Gelatin

Gelatin is a soluble protein formed by the irreversible partial hydrolysis of collagen and can be obtained from fish, insects, and the skin of land animals. Gelatin is a high molecular weight polydisperse peptide that is widely used in food and biomedicine due to its gelling and thickening properties. Chemical and physical crosslinking methods can be used to make gelatin hydrogels. Gelatin gels have a number of disadvantages, including rapid deterioration, poor mechanical quality, and lack of heat stability [95]. Therefore, gelatin methacrylate (GelMA) and phyllosilicate (LAPONITE)^®^ nanosilicates were developed to form nanocomposite hydrogels with the aim of controlling the release of stem cell secretomes. The results showed that encapsulation of stem cell-derived secretions in nanocomposite hydrogels provides a promising alternative in cardiac tissue regeneration therapy. The cell-derived secretion-derived hydrogel nanocomposite provides a dual-action therapeutic system through its proangiogenic and cardioprotective capabilities [15].

In another study, MSC CM could be encapsulated into nanoparticles and then inserted into a hydrogel to form a composite without losing its functional characteristics. MSC CM nanocomposite hydrogels were prepared using gelatin, hyaluron, and Poly Latic Acid for the regulated release of MSC CM. The biocompatibility of MSC CM hydrogel is indicated by its ability to facilitate MSC adhesion and proliferation, thereby increasing the metabolic activity and potential of MSC as a regenerative drug [85]. For the culture of bone marrow-derived mononuclear cells, electrospun polycaprolactone-gelatin nanofiber matrices (PCG matrix) were used together with growth media mixtures (BM MNCs). As a result, the system generated a mixed cell population consisting mostly of EPCs, with appreciable proportions of MSCs and pericytes. This technique, in particular, makes it possible to trap the cellular secretome in the form of a hydrogel; with this technique, the secretome can be easily extracted and applied directly to the diabetic wound, leading to accelerated wound healing [65].

#### 4.2.7. Polyethylene Glycol (PEG)

Polyethylene glycol (PEG) is also known as polyoxyethylene (POE) or polyethylene oxide (PEO), depending on the synthesis conditions and molecular weight. The Food and Drug Administration (FDA) has certified PEG as a synthetic substance with excellent biocompatibility, low cost, and water solubility. PEG is used in a variety of biomedical applications, from wound healing to drug delivery [96]. The PEG hydrogel is a versatile platform that can be used to deliver chondrocytes and cartilage tissue engineered cells. Chondrocytes encased in this synthetic hydrogel contain cartilage-specific ECM components, the number of which continues to increase over time. The chondrocyte secretome consists of proteins that have been found in chondrocyte exosomes, as well as proteins that are important for the development and organization of the cartilage ECM. These findings imply that PEG hydrogels can be designed for cartilage tissue engineering with various initial qualities and loading settings, without significantly affecting chondrocyte secretion. However, the loading and/or structure of the hydrogel may affect the concentration of released protein, which requires further investigation [74].

#### 4.2.8. Poly(N-isopropylacrylamide) (pNIPAm)

In recent years, polyacrylamide (PAm)-based hydrogels have been widely used in the pharmaceutical, drug delivery, and biosensor liquid sectors. One of the main characteristics that distinguishes PAm from other materials is its unique and adjustable mechanical properties: the hydrogel strength can be adjusted from less than 1000 pa to several Mpa. Nonetheless, PAm has low toxicity and weak cell adherence, both of which are problems in biomedical applications. PAm-based hydrogels require the integration of natural ingredients (e.g., alginate and collagen) and peptide conjugates (e.g., PEG) [97,98]. Poly(N-isopropylacrylamide) (pNIPAm) allows the attachment, development, and spread of cells in certain situations due to the quality of the 37 °C temperature response. The combination of a low molecular weight pNIPAm thermoresponsive polymer conjugated with PEG to make a highly elastic and strong hydrogel. The findings reveal that a long-term injection encapsulation and delivery (SHIELD) depletion hydrogel with a moderate stiffness range (200–400 Pa) can dramatically increase the angiogenic capacity of human adipose stem cells (hASC), increasing therapeutic efficacy, while reducing cell counts. Due to the precise and reversible peptide bond, all mixtures form a weak physical network ex vivo within seconds. Increasing the temperature to 37 °C resulted in the development of a peptide-PEG-PNIPAm copolymer reinforcement network with regulated stiffness [69]. A functionalized NIPAAm-based hydrogel with thermally adjustable degradation and injection properties was recently presented in a study. The rate of degradation of the NIPAAm-based hydrogel can be easily regulated by using the NIPAAm hydrogel with methacrylic acid monomer [68] and showed that the low stiffness of the material can significantly increase the expression of pro-angiogenic transcripts, such as VEGF and bFGF, in ADMSCs to some extent, higher than conventional 3D spheroid culture MSCs, using a fibronectin-conjugated polyacrylamide hydrogel (FN-PAAm) with an adjustable Young’s modulus (E = 0.15 kPa). CM obtained from ADMSCs cultured at 0.15 kPa FN-PAAm substantially promoted in vitro and ex ovo vascularization events. These findings emphasize the need to identify important ingredient features that could enable the reprograming of cellular redox signaling for enhanced MSC-based secretome regenerative therapy.

#### 4.2.9. Poly(lactic acid-co-glycolic) (PLGA)

The encapsulation of the ECM in biodegradable and biocompatible nano or micro carriers allows it to maintain its structure and integrity, thereby maintaining biological activity. The encapsulation of BDNF in microparticles made of biocompatible poly (lactic acid-co-glycolic) (PLGA) is a strategy to protect BDNF from the environment and provide continuous delivery at the injection site, because the released BDNF undergoes increased secretion of growth factors by cells and potentially has a synergistic action. However, the efficient and sustainable release of protein from FDA-approved PLGA-based scaffolds remains a challenge due to protein instability during the formulation process. Bioactive BDNF combined with nanoprecipitation silanized-hydroxypropyl methylcellulose (Si-HPMC) hydrogels encapsulated in PAm made from polymers that are more hydrophilic than PLGA, such as the polymeric PLGA-poloxamer(P188)-PLGA triblock, are also believed to maintain the structure and biological activity of BDNF [99].

#### 4.2.10. Others

Silanized-hydroxypropyl methylcellulose (Si-HPMC) is a non-toxic injectable hydrogel with moderate swelling qualities that forms a gel in situ at physiological pH. Si-HPMC hydrogel can be used on a continuous release basis as a therapy for cell delivery, directing neurodevelopment, and enhancing its protective/reparative properties [60]. Polycaprolactone (PC) hydrogels have been widely used in biomedicine for decades as a biomaterial for implants in sutures and wound dressings, cardiovascular tissue engineering, nerve regeneration, and bone tissue engineering [100]. Other studies have shown that the use of PC provides the easy absorption of secretome in a hydrogel-like consistency, without reducing the concentration and activity of the active components, making it suitable for accelerating wound healing [65]. The development of MSCs along osteogenic lineages can also be facilitated by silk fibroin (SF) as a scaffold material for bone tissue fabrication. SF is a fibrous protein, mainly produced by spiders and silkworms [101]. SF hydrogels have been shown to be biodegradable and biocompatible, and they can also be used as vehicles for the encapsulation and delivery of cells and bioactive compounds. The bioactivity of the secretome-loaded gel was validated in vitro, and the release process was maintained. It was found that transplantation of the secretome-containing SF hydrogel resulted in a clear reversal of the detrimental effects of osteoporosis [66].

## 5. Author’s Perspective

The development and improvement of methodologies and technologies in MSC secretome culture, as well as a global understanding of the secretome components, will be necessary to promote secretome-based therapies and determine their safety and efficacy [102]. Recently, a pilot study proposed a good manufacturing protocol (GMP) to transform MSC secretome into a pharmaceutical product, by combining an integrative manner ultrafiltration and freeze-drying techniques, the establishment, validation, and consequent use of a GMP [1]. Clinical practice MSC secretome can be reached by expanding MSCs in defined GMP culture conditions that are reproducible, scalable, and well-controlled aiming to limit heterogeneity and enhance the predictability of secretome-derived products in terms of composition and function [59]. The use of biomaterials has also been looking at an add-on to modulate secretome production, and even as a way to deliver it [69]. However, although the potential of this is enormous. the challenge to prove it remains [103]. Furthermore, the selection of hydrogels is a very important indicator in the biomedical industry, due to the need for non-invasive therapies. Hydrogels can be made and developed by combining natural and synthetic polymers with flexible chemical structures and mechanical properties, as well as changeable degradation rates, like ECM elements [27]. Combining biomaterial with MSC secretome in the recovery/regeneration of the wounded uterus in a mouse model with Asherman’s syndrome enhanced endometrial morphology and fertility, while avoiding the tumorigenic risks associated with cell treatment. HA biomaterials have been shown to be promising candidates as optimum carriers in controlled-release drug delivery systems for intrauterine administration, speeding up the repair process because of their strong antiadhesive, stability, and biodegradability. As a result, future biomaterial research will concentrate on a more in-depth investigation of the material properties of the hydrogels used to fabricate “seamless” hydrogels that can be adapted, while continuing to mimic an extracellular environment to improve the therapeutic platform using the secretome [80,104].

## 6. Conclusions

In various biomedical applications, several types of polymeric hydrogel-based systems have been shown to localize delivery and enhance the therapeutic efficacy of MSC secretomes. However, only a few studies, particularly in vivo, have fully studied the loading and release rates of MSC secretome. Hydrogels are suitable carriers for stem cells in a variety of biomedical applications due to their physical qualities similar to native ECMs. In addition, hydrogels are known to regulate the fate of stem cells to release their bioactive factors in order to produce a secretome that is more precise and efficient in its therapeutic effects, such as accelerating tissue and organ regeneration. The quality of the polymeric hydrogel material can be modified based on the characteristics of the polymer because it affects the hydrogel stiffness, biodegradability, and biocompatibility. The modification of the properties of polymeric hydrogels is very important to study and should be considered in particular because hydrogels are known to interact with the environment in which they are placed. In order to gain a better knowledge of the mechanisms underlying the action and optimize the therapeutic potential, in-depth research on the release profile of MSC secretome carrier-based with suitable controls in biomedical applications is required.

## Figures and Tables

**Table 1:** Studies on the use of mesenchymal stem cell secretomes in biomedical applications.

Source Secretome	Bioactive Molecules	Biomedical Apps	References
SD-MSCs	VEGF, MMP2, PDGFR-β, FN1, DCN, Collagen 1, Collagen 3, TGF-β1, and Ang-1	Skin regeneration after injury	[7,58]
hASCs	collagen XVIII and HGF	Treating myocardial infarction	[15]
RAA-MSCs, WJMSCs	BDNF, NGF, VEGF, GDNF, FGF-2, IGF-1, HGF	Parkinson’s disease	[19,59]
ECM	VEGF-A and angiopoietin- 2	Growing ovarian follicles	[22]
BMMSCs	EGF, IGF, FGF, IGFBP	Asherman’s Syndrome (USA)	[24]
BMMSCs	BDNF, b-NGF, SCF, HGF, LIF, PlGF-1, SDF-1α, VEGF-A& D	Treatment of neurological disorders	[27,60]
hUCESCs, hASCs	EGFR, FGF 4 and 9, IL–6, VEGFD, ICAM3, MCP3, MIF, sgp130	Cancer	[49,61]
BMMSCs, hUCESCs	MMP 1 and 2, FGF 6 and 7, urokinase receptor, and HGF	Corneal epithelial wound healing	[17,62]
EPCs & renal-MSCs	IL-10, IL-4, IL-6, and KC	Acute kidney injury	[63]
ADSCs, EPCs	IL-6, Ang-1, GM-CSF and (PG)E2	Diabetic wound repair	[64,65]
hUCMSCs	TGF-β, EGF, FGF, IGF-1, VEGF	Osteoporosis therapy	[66]
MSCs	VEGF and FGF	Heart tissue repair	[67]
ADMSCs	VEGF, PEDF and PDGF	Angiogenesis therapy	[68,69,70]
hMSCs & rACs	TGF-β, BMPs and IGF-1	Osteoarthritis clinical therapy	[71,72]
hMSCs, ASCs	TGF-β3, TGF-β1, IL-6, and IL-8	Cartilage repair	[73,74,75]
ADMSCs	VEGF-A and -D	Bladder regeneration	[76]
UCMSCs	Milk fat globule-EGF factor 8 (MFGE8)	Liver fibrosis	[21]

**Table 2:** Some hydrogel-forming polymers for MSC secretome delivery.

Polymer	MSC Secretome Source	Type of Hydrogel	References
Carrageenan or PVA	SDMSCs	Hydrogel	[7]
Bovine collagen (COLL 1) with HA or PEG	RAA-MSCs		[19]
Alginate	Extracellular matrix		[22]
Hyaluronic acid	hBMSCs		[24]
Gellan gum and glucuronic acid	BMMSCs		[27]
Hyaluronic acid	EPCs, renal MSCs		[63]
Si-HPMC and PLGA	MIAMI		[60]
Silk fibroin	hUCMSCs		[66]
FN-PAAm	ADMSCs		[68]
Alginate	hMSC, rMSCs		[73]
HA and Chondroitin sulfate	BMMSCs	Viscoelastic gel	[62]
Gelatin and phyllosilicate (LAPONITE)^®^	hASCs	Nanocomposite injectable hydrogel	[15]
Phyllosilicate (LAPONITE)^®^ nanosilicates and collagen	MSCs	Nanocomposite hydrogels	[67]
Gelatin and HA	BM-MSCs		[85]
PEG and pNIPAm	ASCs	Encapsulation in hydrogels	[69]
Alginate	hMSCs, rACs		[71]
PEG	Chondrocyte secretome		[74]
HA and gellan gum	hASCs	Spongy-like hydrogels	[64]
Polycaprolactone (PC) and gelatin	EPCs	3D electrospun nanofiber	[65]
Hyaluronic acid	ASCs	Micro-molded non-adhesive hydrogel	[75]
Collagen	pADSC and SMC	Compressed hydrogel	[76]

## Data Availability

All data included in the article.

## Abbreviations

ECM	Extracellular matrix
MSCs	Mesenchymal stem cells
hMSCs	Human mesenchymal stem cells
hASCs	Human adipose stem cells
BMMSCs	Bone marrow-derived mesenchymal stem cells
hUCMSCs	Human umbilical cord-derived mesenchymal stem cells
ADMSCs	Adipose-derived mesenchymal stem cells
SDMSCs	Skin-derived mesenchymal stem cells
pADSC	SMC-like adipose-derived stem cells
WJMSCs	Wharton’s Jelly mesenchymal stem cells
hUCESCs	Human uterine cervical stem cells
UCPVCs	Umbilical cord perivascular cells
PDLSCs	Periodontal ligament stem cells
EPCs	Endothelial progenitor cells
SMC	Smooth muscle cells
RAA-MSCs	Rat Adipose tissue-derived mesenchymal stem cells
rACs	Rabbit articular chondrocytes
HA	Hyaluronic Acid
CG	Carrageenan
RGD	Arginyl glycyl aspartic acid peptide
NIPAAm	N-Isopropylacrylamide
SHIELD	Shearthinning hidrogels for injectable encapsulation and long-term delivery
MMP	Matrix metalloproteinase
FN	Fibronectin
DCN	Decorin
PDGFR β	Platelet-derived growth factor receptor β
Ang-1	Angiotensin-1
GDNF	Glial cell line-derived neurotrophic factor
FGF	Fibroblast growth factor
EGF	Epidermal growth factor
IGFBP	Insulin-like growth factor binding protein
SGF	Secrete growth factors
SCF	Stem cell factor
LIF	Leukemia inhibitory factor
SDF	Stromal cell-derived factor
PlGF-1	Placenta growth factor-1
ICAM3	Intercellular adhesion molecule 3
MCP3, also called CCL7	Monocyte-specific chemokine 3
MIF	Migration inhibitory factor
sgp130	Soluble glycoprotein 130
KC	Keratinocyte-derivedchemokine
GM-CSF	Granulocyte macrophage colony-stimulating factor and
(PG)E2	Prostaglandin
PEDF	Pigment epithelium-derived factor
